# Glutathione revisited: a better scavenger than previously thought

**DOI:** 10.3389/fphar.2014.00260

**Published:** 2014-11-27

**Authors:** Guido R. M. M. Haenen, Aalt Bast

**Affiliations:** Department of Toxicology, Faculty of Health, Medicine and Life Sciences, Maastricht UniversityMaastricht, Netherlands

**Keywords:** glutathione, antioxidant, hypochlorous acid, alpha 1-antitrypsin, acetylcholinesterase, rate constant

## Abstract

Glutathione (GSH) is the classical example of a scavenging antioxidant. It forms the first line of defense and efficiently scavenges reactive species, e.g., hypochlorous acid (HOCl), before they inflict damage to biomolecules. Scavenging antioxidant activity is best established in competition assays (that closely mimics molecular mechanism of the biological effect). In this type of assay, the antioxidant competes with a molecule that functions as an easy read-out detector for a reactive species. It is generally assumed that the scavenging antioxidant activity reflects the reaction rate constant of the antioxidant with the reactive species (k_a_). However, critical appraisal of several competition assays of GSH with HOCl as reactive species, reveals that k_a_ does not determine the scavenging antioxidant activity. Assays using acetylcholine esterase, alpha1-antiprotease, methionine, and albumin as detector are compared. The total number of molecules of the reactive species scavenged by GSH plus that by partially oxidized forms of the GSH, reflect the scavenging activity of GSH. The contribution of the partially oxidized forms of GSH depends on the reactivity of the competing molecule. In several assays the partially oxidized forms of GSH have a substantial contribution to the scavenging activity of GSH. In contrast to the prevailing perception, not the reaction rate but rather the total number of molecules of the reactive species scavenged reflects the true scavenging activity of an antioxidant like GSH.

## INTRODUCTION

Many diseases are associated with the production of reactive oxidizing species that damage physiologically essential molecules. The classical view is that antioxidants scavenge these reactive oxidizing molecules and thus offer protection against disease. Antioxidants can be either enzymatic or non-enzymatic in nature. Together they form an elaborate web and protect the organism from widespread oxidative damage. Moreover, non-enzymatic antioxidants which are frequently identified in the diet are major constituents of food supplements or are administered as drugs. Antioxidant activity of compounds is commonly determined by means of competition assays. Based on these *in vitro* assays effective antioxidants are selected for further development and for further human use. Recently, doubts have been raised as to the *in vivo* relevance of antioxidants in disease protection in general. Several misconceptions on antioxidants have been uncovered (Bast and Haenen, 2013).

What thus far undisputedly remained is the competition assay as an unquestionable way to identify and categorize either endogenous or exogenous antioxidants. Similarly, the antioxidant action of cellular glutathione (GSH) in competitive protection against redox damage is also undeniable. GSH competes with other cellular redox active biomolecules for oxidizing reactive species and thus is a vital cellular protagonist.

In competition assays, the antioxidant (A) competes with a detector molecule (D) for the reactive oxidizing species (R). In other words, the antioxidant (A) protects the detector molecule (D) by scavenging the reactive species (R). The detector molecule (D) of course mimics the biomolecule which can be affected by R

$$R\,\;\;\;\;\;\begin{array}{l} +A\overset{k_{a}}{\to }oxidized\,\;\;\;antioxidant\\ +D\overset{k_{d}}{\to }oxidient\,\;\;\det ector. \end{array}$$

From this scheme it can be derived that the rate of radical scavenging by A is proportional to the rate constant k_a_ of the reaction of A with R and the concentration of A.

Comparison and critical evaluation of various competition assays revealed that not the reaction rate constant of GSH with the reactive species determines its scavenging antioxidant activity, but rather the total number of molecules of the reactive species scavenged, reflect the true scavenging activity of the antioxidant GSH.

## MATERIALS AND METHODS

### MATERIALS

Reduced GSH, sodium hypochlorite, L-methionine, and 1,4-dithiotreitol were purchased from Sigma-Aldrich (St. Louis, MO, USA). All other chemicals were of analytical grade. All experiments were performed in 145 mM potassium phosphate buffer pH 7.4.

### METHODS

#### Determination of stoichiometry and capacity

The stoichiometry of the antioxidant (here either GSH or 1,4-dithiothreitol) is defined as the number of molecules of reactive species that react directly with one molecule of antioxidant. The observed stoichiometry was quantified by determining the concentration of the antioxidant immediately (within 30 s) after addition of a relatively small amount of HOCl. The remaining concentration of the antioxidant was plotted against the initial concentration of HOCl and the data were fitted linearly. The intercept of the linear fit with the x-axis was divided by the initial concentration of antioxidant to obtain the observed stoichiometry. It should be noted that the observed stoichiometry, determined by this procedure, includes the products that react much faster with the reactive species than does the parent compound and might deviate from the actual stoichiometry.

**FIGURE 1:**
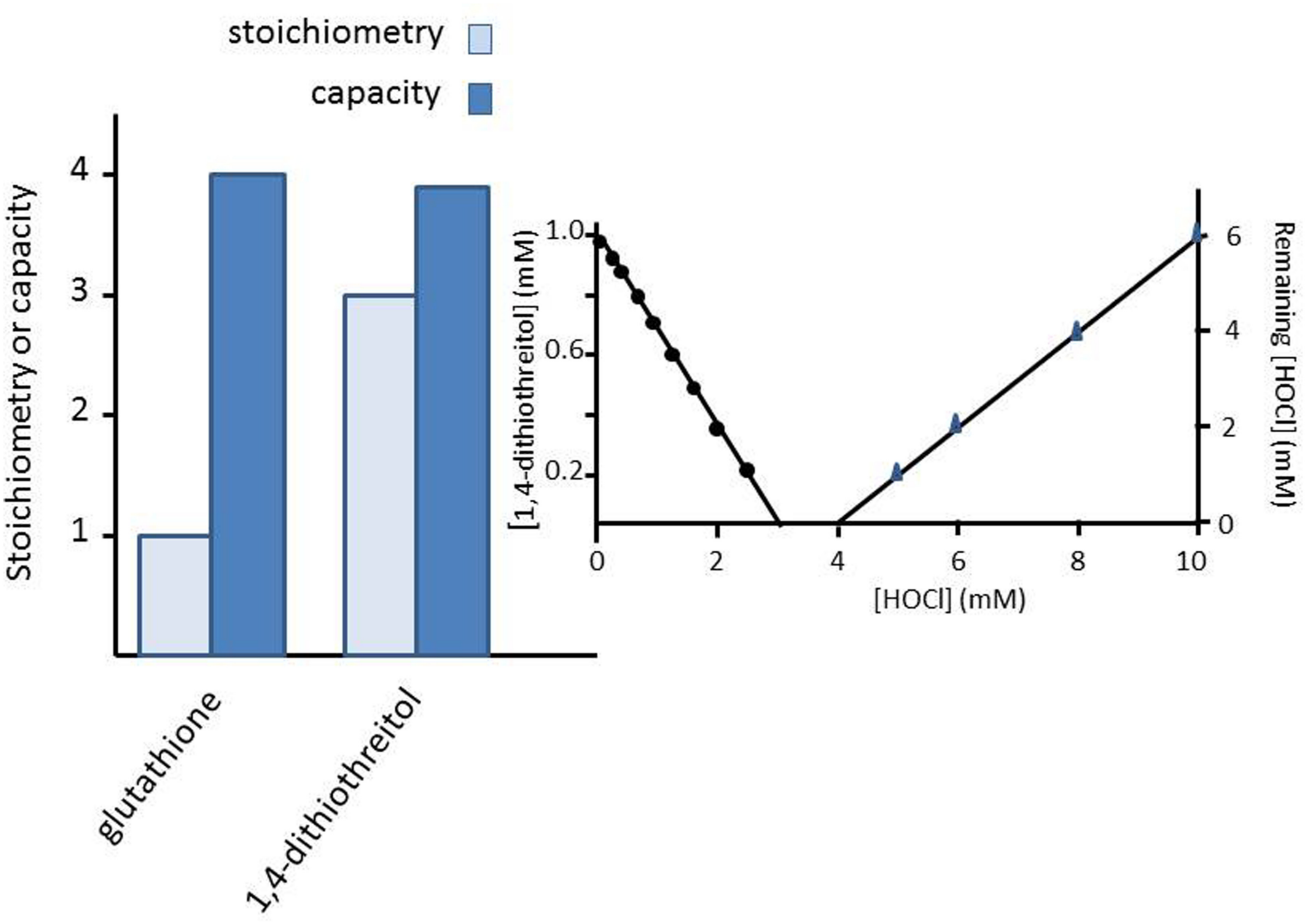
**The stoichiometry and the capacity of the reaction of either 1 mM of glutathione (GSH) or 1 mM of 1,4-dithiothreitol with increasing concentrations of HOCl (up to 10 mM).** The inset shows the bar diagrams are obtained for 1,4-dithiothreitol. Note that three molecules of 1,4-dithiothreitol are consumed, but only after the consumption of almost four molecules HOCl is detected. This leads to a stoichiometry value of 3 and a capacity of 4. Similarly, GSH has a stoichiometry of 1 and a capacity of 4.

The total scavenging capacity is defined as the number of molecules of HOCl that are scavenged by the antioxidant within a certain time span, when an excess of HOCl is added to the antioxidant. The total scavenging capacity is the sum of the stoichiometry of the parent antioxidant, of the first product and of all other products formed in subsequent reactions. The total scavenging capacity of the antioxidants (GSH or 1,4-dithiothreitol) was determined by adding HOCl in a concentration range of 0–10 mM to a fixed concentration of the antioxidant of 1 mM. Thirty seconds after adding HOCl, the concentration of remaining HOCl that was in excess, was determined spectrophotometrically. The remaining concentration of HOCl was plotted against the initial concentration of reactive species and the data was fitted linearly. The intercept with the x-axis was divided by the initial concentration of the antioxidant to obtain the total scavenging capacity. The procedure to determine the observed stoichiometry and total scavenging capacity of 1,4-dithiothreitol is illustrated in the inset of **Figure 1**.

The chemical nature of the conducted experiments results in very small errors, which have not been shown for sake of clarity.

#### Competition assays

In the competition assay with methionine as detector in a final concentration of 1 mM of methionine was used. Different concentrations of the GSH were added to the methionine containing solution. The mixture of the detector (methionine) and the antioxidant (GSH) was pre-incubated at 37∘C for 5 min. During rigorous mixing, HOCl was added (final concentration of 0.63 mM). After completion of the reaction, the remaining amount of the antioxidant GSH was determined (Peskin and Winterbourn, 2001). The EC_50_ is defined as the concentration of antioxidant that reduces the signal produced by the detector by 50 percent compared to the signal obtained in the absence of the antioxidant.

The advantage of the HOCl scavenging assay is that all of the reactive species are scavenged by either the detector or antioxidant, and therefore there is no systematic underestimation of antioxidant activity as in numerous other assays.

## RESULTS

Glutathione is an important antioxidant and its protective action in various lung diseases is certain. In fact in several large multi-center clinical trials the GSH precursor *N*-acetylcysteine was used in attempts to increase the lung epithelial levels of GSH. For example in COPD patients *N*-acetylcysteine was shown to decrease the intensity and frequency of exacerbations (Decramer et al., 2005). In idiopathic pulmonary fibrosis patients three times 600 mg daily resulted in less worsening of the lung function (Demedts et al., 2005).

Damage to numerous vital enzymes in the lung by oxidants has been associated with various lung diseases. GSH protects. One of the oxidants involved is HOCl. This oxidant is generated from neutrophils by the enzyme myeloperoxidase (Park et al., 2013)

Glutathione is known to react with HOCl. We therefore investigated and re-evaluated GSH in various competition assays to establish its efficacy to protect physiologically critical enzymes in lung pathology which are subjected to the oxidant HOCl.

Acetylcholinesterase is among others of importance in lung physiology, because it removes the smooth muscle contracting acetylcholine. Damage by HOCl to acetylcholinesterase will lead to lung smooth muscle contraction and hyperreactivity by disturbing the cholinergic homeostasis (den Hartog et al., 2002). The EC_50_ value of GSH against damage by 1 μM HOCl on acetylcholinesterase is 0.2 μM (den Hartog et al., 2002).

Alpha-1 antiprotease (or α_1_-antitrypsin or protease inhibitor) is another important enzyme in lung pathophysiology since it inactivates elastase. The latter is released during inflammation by neutrophilic granulocytes and breaks down connective tissue fibers elastin. Oxidative damage of alpha-1 antiprotease has been associated with the lung disease emphysema.

In a competition assay in which the protection by GSH is characterized, the EC50 value of GSH against damage by 100 μM HOCl on alpha-1 antiprotease is 20 μM.

The amino acid which is critical in the function of alpha-1 antiprotease is methionine (Taggart et al., 2000). We therefore also investigated the protection of GSH on the HOCl mediated oxidation of methionine. The EC_50_ value of GSH against oxidation by 630 μM HOCl of methionine is 520 μM.

Literature data show that albumin oxidation by 1600 μM HOCl is inhibited by GSH with an EC_50_ value of 200 μM (Gatto et al., 2002). With lower levels of HOCl (200 μM) led to an EC_50_ of GSH of 38 μM (Yan et al., 1996).

In an effort to understand these striking differences in EC50 values of the protective action of GSH on HOCl-induced oxidative damage (as summarized in **Table 1**), we computed the reaction of GSH with HOCl. It seemed that stoichiometrically one molecule of GSH is rapidly consumed by reaction with one molecule HOCl but the total HOCl scavenging capacity of GSH amounts to 4, i.e., four molecules of HOCl are scavenged in total by one molecule of GSH (**Figure 1**).

**Table 1:** The antioxidant activity of glutathione (GSH) indicated by its EC_50_ value in various completion assays.

	EC_50_-value (μM)	[HOCI] (μM)	Reference
Acetylcholine esterase	0.2	1	den Hartog et al. (2002)
Alpha1-antiprotease	20	50	Haenen and Bast (1991)
Methionine	520	630	This paper
Albumin	38	200	Yan et al. (1996)
	200	1600	Gatto et al. (2002)

*Protection by GSH on HOCl-induced damage. The initial HOCl concentration is depicted as [HOCl]*.

For comparison, 1,4-dithiothreitol has a stoichiometry of three and a total capacity of almost four (**Figure 1**).

## DISCUSSION

Competition experiments are frequently used to establish the protection of antioxidants against oxidative damage of important oxidative sensitive biological targets [detectors, (D)]. The competition experiments are relatively easy to perform and frequently give rise to far reaching conclusions on the value of antioxidants. These seemingly uncomplicated experiments have an outcome that is, however, more complex to interpret.

We already published that the initial concentration of the reactive species (R) can influence the observed activity of an antioxidant (Balk et al., 2009).

It can be derived that the EC_50_ value of antioxidants is linked to the rate constant (k_a_) of the antioxidant with the reactive species, as follows:

$$\begin{array}{cc} k_{a} & =(k_{d}/{EC}_{50}){\left[D\right]}_{0}\\ {\begin{array}{c} EC \end{array}}_{50} & =(k_{d}/k_{a}){\left[D\right]}_{0}. \end{array}$$

In this derivation it is assumed that the concentration of the detector [D] does not vary during the experiment and equals the initial concentration [D]_0_, likewise it is assumed that [A] remains constant during the experiment and equals the initial antioxidant concentration [A]_0_. We published earlier that concentrations of [D] and [A] do not decrease to the same extent in a competition experiment (Balk et al., 2009). This greatly affects the outcome of competition experiments.

The current results show that even under the above strict conditions it is sometimes not just the reaction rate constant k_a_ of the antioxidant with the oxidant that governs the antioxidant protection of biological targets. This is clearly presented in **Figure 1**. Stoichiometrically, one molecule of GSH rapidly reacts with HOCl, but at the same time in total four molecules of HOCl are scavenged by one molecule of GSH. For comparison, 1,4-dithiothreitol has a stoichiometry of three and has a total capacity of almost four molecules (**Figure 1**).

Glutathione has a total HOCl scavenging capacity of four. It appears that stoichiometrically one molecule of GSH is rapidly consumed by reaction with one molecule HOCl but the total HOCl scavenging capacity of GSH amounts to 4, i.e., four molecules of HOCl are scavenged in total by one molecule of GSH (**Figure 1**).

**FIGURE 2:**
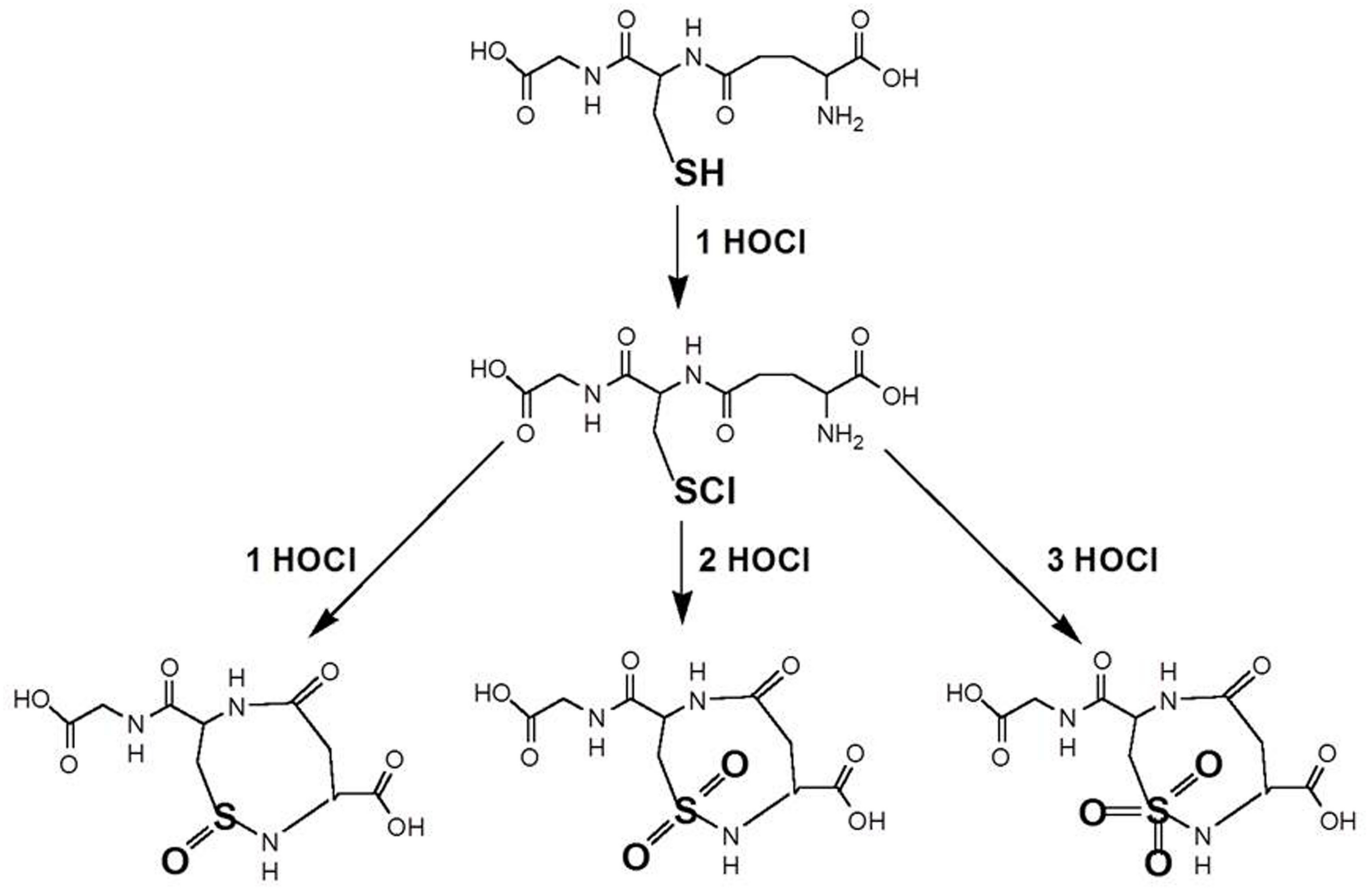
**Putative reaction scheme of GSH with OCl^-^ (after Winterbourn and Brennan, 1997).** The initial reaction is the formation of glutathione sulfenyl chloride (GSCl). Further suggested products are depicted as oxidized condensation products of the sulfonyl chloride with the amino group of the glutamyl residue of GSH.

It is suggested that GSH reacts with HOCl in a step wise fashion to form four products. Interestingly, several higher oxidation products have been suggested to occur before (Winterbourn and Brennan, 1997). It might be proposed that the initial reaction is GSH + OCl^-^ → OH^-^ + GSCl (**Figure 2**). The observed stoichiometry of four indicates that the fourth product has a slower reaction rate with HOCl than GSH. It is not uncommon that oxidation products of antioxidants are more reactive than the parent antioxidant as we have reported earlier (Arts et al., 2004).

Interestingly several other reaction products have indeed been suggested, including a GSH sulfonamide (Pullard et al., 2001; **Figure 2**).

Apparently, the products of the reaction of GSH with HOCl also add to the total antioxidant activity of GSH. The contribution of these higher oxidation products of GSH can be estimated from the observed antioxidant activity.

If one antioxidant molecule would scavenge one HOCl then would the EC_50_ value be half of the initial HOCl concentration ([HOCl]_0_) and thus would in that case the ratio [HOCl]_0_/EC_50_ be 2. In other words {[HOCl]_0_/EC_50_}× 1/2 will be 1.

In the scenario that the oxidation products of the antioxidant contribute to the antioxidant activity the {[HOCl]_0_/EC_50_} × 1/2 will be higher than 1. This ratio has been calculated (**Table 2**) for GSH protection of HOCl-induced damage of acetylcholine esterase (2.5), alpha-1 antiprotease (1.25), methionine (0.6), and albumin (4). The ratio {[HOCl]_0_/EC_50_} × 1/2 gives the average number of HOCl molecules scavenged by the antioxidant GSH at EC50 conditions. Comparing the extremes (methionine versus albumin as target or detector) shows that there is a large difference in the contribution of the oxidation products of GSH in the overall protection against the HOCl-induced damage to either methionine or albumin by GSH. This can be explained by the difference in reaction rate between HOCl with methionine (which is relatively high) and the rate between HOCl with albumin (which is relatively low).

**Table 2:** The genuine scavenging activity of glutathione (GSH) which is obtained from by {[HOCl]_0_/EC_50_} × 1/2.

	Genuine scavenging activity
Acetylcholine esterase	2.5
Alpha1-antiprotease	1.25
Methionine	0.6
Albumin	2.63
	4

Also in the protection by GSH against HOCl induced damage to acetylcholine esterase or alpha-1-antiprotease, the reaction products of GSH contribute to the overall activity of GSH.

Evidently, the reaction rate constant k_a_ determines whether scavenging occurs. However, the genuine scavenging activity is primarily determined by the number of oxidant molecules (in this study HOCl) scavenged. GSH is not just a good biological antioxidant because of its reaction rate with HOCl which is generated during inflammations by myeloperoxidase but rather because the higher oxidation products generated are effective antioxidants. The amount of reactive HOCl molecules scavenged by GSH rather than its initial reaction rate marks GSH a better antioxidant than previously thought!

## Conflict of Interest Statement

The authors declare that the research was conducted in the absence of any commercial or financial relationships that could be construed as a potential conflict of interest.
